# 

**Published:** 1907-04-27

**Authors:** 


### NOTES, QUESTIONS, AND COMMENTS.

The editor of the Resident Medical Officers' Department of The Hospital invites contributions to this column both from resident medical officers and from other members of the profession. Short articles, notes, queries, or suggestions bearing upon this branch of medical practice will always receive careful consideration, and those that are suitable will be published in due course. Correspondence, short and to the point, is particularly invited, as by this means the value of the R.M.O. Department will be greatly increased.

Articles, whether in the form of letters to the editor or otherwise, should in no case exceed 600 words in length, and should, if possible, be shorter than this. Postcards with short notes, questions, or notifications of recent hospital appointments will be welcomed.

All communications for this column must be guaranteed by the name of the writer, which will not be published unless he expressly wishes it, and should bear the words "R.M.O. Department" on the envelope or the address side of the postcard.

Items of news from the resident officers' quarters of the principal hospitals, infirmaries, and asylums throughout the kingdom are invited, and, when of sufficient general interest, will be published.

The editor will also be pleased to receive and reply to confidential communications from resident medical officers and to consider any suggestions that may be made for the improvement of this department of The Hospital.

### GENERAL PRACTITIONERS' CONTRIBUTIONS.

**Important.**

We propose to devote a special page to General Practitioners' Contributions. We therefore invite from practitioners contributions based upon their experience in the management of cases, and in the treatment and diagnosis of disease; especially shall we be prepared to welcome articles dealing, practically, with treatment, and with the use and value of new remedies and methods.

No article should exceed 1,100 words in length, and, if accepted, one guinea will be paid to the writer after publication. Each communication should be accompanied by a stamped directed envelope for the return of the MS. if found unsuitable. See coupon on Special Supplement.

**The Relaxations of Medical Men.**

We shall also be glad to pay for accepted contributions, from any member of the profession, on the subject of the relaxations of practitioners. This opens up a wide field, as it includes natural history, photography, sport, indoor recreations, and motoring. Whenever possible, original illustrations and photographs should be sent with the MS.

**Suggestions Invited.**

The Editor will welcome suggestions for the establishment of any new section in The Hospital, and will be glad to supply information on any subject of interest or importance to members of the profession in any part of the world.

### NOTICES AND ANSWERS TO CORRESPONDENCE.

All MSS., letters, books for review, and other matters intended for the Editor, should be addressed to:—

The Editor,
The Hospital Building,
28 and 29 Southampton Street,
Strand, London, W.C.

**Contributions.**

Contributions should be written, or preferably typed, on one side of the paper only, and all articles sent in are accepted upon the distinct understanding that they are forwarded to The Hospital only.

**Correspondence.**

Correspondence on all subjects is invited, but no communication can be entertained if the name and address of the correspondent are not given as a guarantee of good faith, but not necessarily for publication. All correspondents should write on one side of the paper only.

**Books for Review.**

Publishers are particularly requested to send advance proofs of any new books of importance, whenever possible, as the Editor has made arrangements to publish immediate reviews, and on a new plan.

### BUSINESS NOTICES.

Letters relating to the Publishing, Sale and Advertisement Departments must be addressed to the Manager (*not to the Editor*):—

The Manager,
The Hospital Building,
28 and 29 Southampton Street,
Strand, London, W.C.

**Annual Subscriptions.**

The rate of annual subscriptions to The Hospital, either direct from the Office or through agents, is:—

For the United Kingdom ... ... 13s. a year
To the Colonies and Abroad ... 17s. a year inclusive of postage in each case.

**Subscriptions.**

Subscriptions are payable in advance. Cheques and Postal Orders should be made payable to:—

The Scientific Press, Ltd., and should be crossed: London and County Banking Co., Ltd.

**Cases for Binding.**

Cases for binding half-yearly volumes of The Hospital may be obtained from the Manager, price 2s., post free. The Index to Vol. xli. is now ready, price $3\frac{1}{2}$d., post free. The monthly part for March is also ready, price 1s., by post 1s. 3d.

